# Proinsulin C-peptide - A Consensus Statement

**DOI:** 10.1155/EDR.2001.145

**Published:** 2001

**Authors:** Anders A. F. Sima, George Grunberger, Hans Jörnvall, John Wahren, The C-Peptide Study Group

**Affiliations:** 1 Department of Pathology Wayne State University Detroit Michigan USA; 2 Department of Neurology Wayne State University Detroit Michigan USA; 3 Department of lnternal Medicine Wayne State University Detroit Michigan USA; 4 Morris Hood Jr. Diabetes Center Wayne State University Detroit Michigan USA; 5 Center for Molecular Medicine and Genetics Wayne State University Detroit Michigan USA; 6 Department of Medical Biochemistry and Biophysics Section of Clinical Physiology Karolinska Institutet Stockholm Sweden; 7 Department of Surgical Sciences Section of Clinical Physiology Karolinska Institutet Stockholm Sweden

## Abstract

In recent years the physiological role of the proinsulin C-peptide has received increasing attention, focusing on the potential therapeutic value of C-peptide replacement in preventing and ameliorating type 1 diabetic complications. In order to consolidate these new data and to identify the immediate directions of C-peptide research and its clinical usefulness, an International Symposium was held in Detroit, Michigan, on October 20–21, 2000, under the auspices of the Wayne State University/Morris Hood Jr. Comprehensive Diabetes Center. In this communication, we review the cellular, physiological and clinical effects of C-peptide replacement in animal models and in patients with type 1 diabetes. Finally, recommendations are presented as to the most urgent studies that should be pursued to further establish the biological action of C-peptide and its therapeutic value.


Int. Jnl. Experimental Diab. Res., Vol. 2, pp. 145–151
Reprints available directly from the publisher
Photocopying permitted by license only

© 2001 OPA (Overseas Publishers Association) N.V.
Published by license under
the Harwood Academic Publishers imprint,
part of Gordon and Breach Publishing,
a member of the Taylor & Francis Group.
Printed in the U.S.A.

### Proinsulin C-peptide – A Consensus Statement

ANDERS A. F. SIMA[a,b,d,*], GEORGE GRUNBERGER[c,d,e], HANS JÖRNVALL[f], JOHN WAHREN[g] and The C-peptide Study Group[†]

[a]*Departments of Pathology,* [b]*Neurology,* [c]*Internal Medicine,* [d]*Morris Hood Jr. Diabetes Center,* [e]*Center for Molecular Medicine and Genetics, Wayne State University, Detroit, Michigan, USA;* [f]*Departments of Medical Biochemistry and Biophysics,* [g]*Surgical Sciences, Section of Clinical Physiology, Karolinska Institutet, Stockholm, Sweden*

**In recent years the physiological role of the proinsulin C-peptide has received increasing attention, focusing on the potential therapeutic value of C-peptide replacement in preventing and ameliorating type 1 diabetic complications. In order to consolidate these new data and to identify the immediate directions of C-peptide research and its clinical usefulness, an International Symposium was held in Detroit, Michigan, on October 20–21, 2000, under the auspices of the Wayne State University/Morris Hood Jr. Comprehensive Diabetes Center. In this communication, we review the cellular, physiological and clinical effects of C-peptide replacement in animal models and in patients with type 1 diabetes. Finally, recommendations are presented as to the most urgent studies that should be pursued to further establish the biological action of C-peptide and its therapeutic value.**

### INTRODUCTION

C-peptide fulfills an important function in the biosynthesis of insulin by bringing its A and B chains together. The process of folding and disulfide bond formation, necessary for the generation of bioactive insulin, is thereby facilitated.[1,2] C-peptide is subsequently cleaved from the proinsulin molecule and released to the circulation in amounts equimolar to insulin. Following discovery of the mode of insulin synthesis, several early studies addressed the question of possible insulin-like effects of C-peptide. None were found and it became generally accepted that C-peptide had no other role than its participation in insulin biosynthesis. However, recent reports of cellular binding interactions of C-peptide[3] and the finding of physiological effects in patients with type 1 diabetes, who are C-peptide deficient, have prompted renewed interest in this peptide.[4]

### The C-peptide Molecule

The species variability of the C-peptide molecule is greater than for the surrounding A and B chains of insulin, and for many bioactive peptides, but other hormones, like parathyroid hormone and gastrin-releasing peptide, also show considerable inter-species variability. The largely conserved

*Corresponding author. Tel.: 313 577 1150, Fax: 313 577 0057, e-mail: asima@med.wayne.edu
† The total C-peptide Study Group list is appearing at the end of the article.

mammalian sequence pattern of C-peptides includes four non-consecutive positions with glutamic acid, an interspaced glycine-rich segment, and some further residues. As a result, the C-peptide residue pattern provides a basis for ligand-receptor binding interactions. Their nature is yet unknown, but spacing is obviously important, in a manner like that seen for the major histocompatibility molecules, in which the residue spacing provides sites of defined interactions.[5,6]

**Cellular Interactions**

Measurements using fluorescence correlation spectroscopy have established the occurrence of C-peptide cell membrane interactions.[3] There is now direct evidence for a stereospecific binding of C-peptide in the nanomolar concentration range to surfaces of several cell types, *e.g.*, renal tubular cells, fibroblasts and endothelial cells. The specificity of the binding is demonstrated by competitive displacement of bound C-peptide by excess concentration of native C-peptide and by the inability of both scrambled C-peptide (the same amino acid residues as C-peptide but randomly assembled) and D-C-peptide to displace binding. The COOH-terminal pentapeptide segment is essential for C-peptide binding and may constitute an active site of the molecule.[3] The binding probably involves a G-protein, since pretreatment of cells with pertussis toxin results in loss of binding. The affinity binding constant for C-peptide is of the order of $3.10^9$ $M^{-1}$ and saturation of binding occurs at physiological concentrations (~0.9nM). This finding helps explain why no effects of C-peptide have been observed in healthy subjects or animals–binding site saturation is reached already at physiological C-peptide concentrations. Thus, additional effects cannot be expected from further increases in C-peptide concentrations. C-peptide binding is not displaced by molar excess of insulin, proinsulin, IGF-I, IGF-II, or NPY, nor is insulin bound to cell membranes displaced by C-peptide. However, it cannot be excluded that C-peptide binds to *e.g.*, the insulin receptor, but at a different site than that for insulin itself.

Indeed, recent studies suggest a possible interaction between C-peptide and insulin. Data from studies in isolated human skeletal muscle strips indicate that C-peptide stimulates glucose transport, but *via* a mechanism that is independent of the insulin receptor and of tyrosine kinase activation.[7] On the other hand, in rat L6 myoblasts, physiological concentrations of C-peptide has been shown to autophosphorylate the insulin receptor, stimulate tyrosine kinase, IRS-1 tyrosine phosphorylation, P13 kinase activity, MAPK phosphorylation, p 90 Rsk and GSK-3 phosphorylation.[8] These effects result in glycogen synthesis and amino acid uptake. Similar effects cannot be demonstrated by scrambled C-peptide. The data suggest the possibility that the insulin signaling pathway may be activated by both C-peptide and insulin.[8]

A short term effect of C-peptide on glucose utilization has been demonstrated in type 1 patients with the euglycemic clamp technique. These studies demonstrate a 25% increase when C-peptide was acutely raised to physiological levels, whereas further increases had no additional effect.[9] These findings correlate with measurements of increased glucose uptake by resting and exercising forearm muscle during C-peptide infusion.[10] and increased glucose uptake by muscle-strips from diabetic subjects.[11] However, in patients receiving C-peptide plus insulin for 3 months, blood glucose levels, indices of metabolic control, and insulin doses, were all unchanged as compared to non C-peptide treated controls.[7]

The effects of C-peptide concerning two of the characteristic abnormalities in type 1 diabetes, *i.e.*, impaired $Na^+$, $K^+$-ATPase activity and reduced endothelial function are probably more important than the C-peptide influence on glucose utilization. A first outline of an intracellular signal transduction pattern now emerges. Exposure of cells to C-peptide in physiological concentrations results in activation of a G-protein, a rise in intracellular $Ca^{2+}$-concentration from extracellular sources and subsequent activation of $Ca^{2+}$-dependent protein phosphatase IIB, leading to conversion of inactive phosphorylated $Na^+$, $K^+$-ATPase to its active dephosphorylated form.[12] The COOH-terminal pentapeptide is as effective as the intact C-peptide molecule in stimulating $Na^+$, $K^+$-ATPase activity.[13] In addition, in endothelial cells the rise in $Ca^{2+}$-concentration is accompanied by augmented activity of endothelial nitric oxide synthase (eNOS).[14] Furthermore, several findings suggest that C-peptide may interact with other hormones and growth factors. The effect of C-peptide on $Na^+$,$K^+$-ATPase is potentiated by the presence of sub-threshold concentrations of neuropeptide Y.[12]

**Physiological Effects**

C-peptide has been found to elicit concentration-dependent stimulation of $Na^+$, $K^+$-ATPase activity in a variety of tissues including renal tubular cells,[12] rat sciatic nerve,[15,16] pancreatic islets,[17] granulation tissue[16] and red blood cells.[14,18] C-peptide exerts an ameliorating effect on the impaired deformability of red blood cells from type 1 diabetic patients, which is probably mediated *via* its effects on $Na^+$, $K^+$-ATPase. Further support for the C-peptide effects on $Na^+$, $K^+$-ATPase is provided by its effect on rat sciatic nerve $Na^+$, $K^+$-ATPase in type 1 diabetic BB/Wor-rats treated with C-peptide for 8 months.[15,19] This effect is further substantiated by partial correction of the $Na^+$, $K^+$-ATPase associated defect in nerve conduction velocity and paranodal swelling, secondary to axonal $Na^+$ accumulation.[19] Furthermore, in the C-peptide deficient diabetic BB/Wor-rat model the expression of both the insulin receptor and the IGF-I receptor mRNA and protein in peripheral nerve and brain tissue were normalized by C-peptide replacement,[20,21] and the diabetes-induced hippocampal apoptosis was prevented by C-peptide replacement.[21]

In type 1 diabetic patients, autonomic nerve function as measured by heart rate variability during deep breathing improves after intravenous C-peptide infusion for 3h,[22] and in patients who receive C-peptide for 3 months.[7] A subgroup of the latter patients with signs of sensory neuropathy exhibits improved temperature threshold discrimination after C-peptide replacement for 3 months.[7] None of these effects are seen in patients who receive insulin therapy alone. There is no immediate explanation for the beneficial effects of C-peptide on nerve function and structure. It may be related to a stimulation of nerve $Na^+$, $K^+$-ATPase activity, resulting in improved electrolyte balance and enzyme state. Alternatively, the effects may be a consequence of improved neural blood flow. Several studies demonstrate an effect of C-peptide on NO-release. It stimulates eNOS, causing release of NO from bovine aortic endothelial cells in a concentration-dependent manner, an effect that is abolished by NO-synthase inhibitors.[23] This is in keeping with the finding that C-peptide induces an increase in forearm blood flow in type 1 diabetic patients, which is blocked by a NO-synthase blocker.[24] It is also consistent with the demonstration of a C-peptide concentration-dependent dilatation of rat skeletal muscle arterioles in the presence of insulin.[25]

C-peptide replacement in streptozotocin diabetic rats extending from 120 min to 14 days results in diminished glomerular hyperfiltration, increased functional reserve and reduced urinary albumin extraction.[26,27] In addition, the renal weight tends to be lower and the glomerular volume was significantly smaller in C-peptide treated animals then in non-treated controls.[27] These data have been confirmed in clinical studies in patients with type 1 diabetes. Thus, C-peptide replacement during 3h and during 1 month in young patients with early signs of diabetic nephropathy, *i.e.*, glomerular hyperfiltration but absence of manifest microalbuminuria, was accompanied by reduced glomerular hyperfiltration and decreased filtration fraction.[9,28] Furthermore, in a double-blind, randomized, cross-over study, C-peptide replacement for 3 months in patients with microalbuminuria resulted in a 40% reduction in urinary albumin excretion.[7] It may be hypothesized that C-peptide has the potential, *via* stimulation of $Na^+$, $K^+$-ATPase, to influence membrane permeability of the nephron and to normalize regional blood flow, resulting of improvements in renal function in the diabetic state.

Little information is available regarding the possible influence of C-peptide on retinopathy in type 1 diabetes. However, one study demonstrates diminished leakage of fluorescein across the blood-retinal barrier after 4 weeks of C-peptide replacement in young type 1 patients,[24] an effect possibly related to the ability of C-peptide to stimulate blood flow and increase capillary recruitment.[29]

### Summary

Experimental and clinical data now convincingly demonstrate that C-peptide exerts biological effects of its own. The effects are concentration-dependent, in the low physiological range, and most likely mediated *via* stimulation of $Na^+$, $K^+$-ATPase and eNOS activities. Animal and human data indicate that C-peptide has a preventive and ameliorating effect on the chronic complications in type 1 diabetes particularly as regards diabetic neuropathy and nephropathy. However, its mode of receptor interaction, signaling pathways and potential interaction with insulin or other hormones are still incompletely understood. Therefore, based on the deliberations at this consensus meeting, the C-peptide study group agreed on the following recommendations:

### Recommendations

1. Efforts should be directed to identify and characterize the C-peptide receptor and to explore as to whether C-peptide interacts with other receptors.
2. Further work will be needed to identify signaling pathways utilized by C-peptide.
3. Interactions with other hormones, growth factors and transcription factors need to be explored.
4. Elucidation of the effect of C-peptide on blood flow in the target tissues of diabetic complications should be persued.
5. The anti-apoptotic effect of C-peptide should be examined in different tissues and cell systems.
6. Examination of the effects of C-peptide on free radical formation and scavenging in tissues of diabetic complications should be performed
7. Clinical trials should be conducted with respect to diabetic neuropathy and nephropathy.
8. The effect of C-peptide replacement on the development of diabetic retinopathy should be examined in appropriate animal models.
9. Appropriate delivery systems should be designed for convenient and continuous administration of C-peptide.

### *Acknowledgements*

The consensus conference was supported by grants from: Eli Lilly Inc., Indianapolis, IN, Schwarz Pharma, Monheim, Germany and Smithkline Beecham Pharmaceuticals, Philadelphia, PA.

### C-peptide Study Group

Dr. Alexander Breidenbach
Dept. of Pharmacology/Toxicology
Schwarz Pharma AG
Alfred-Nobel-Str. 10
D-40789 Monheim, Germany
Tel.: + 49 2173 481882
Fax: + 49 2173 481574
e-mail: alexander.breidenbach@schwarzpharma.com

Dr. Karin Ekberg
Karolinska Institutet
Dept of Surgical Sciences
Section of Clinical Physiology
Karolinska Hospital
S-171 76 Stockholm, Sweden
Tel.: + 46 8 5177 5154
Fax: + 46 8 32 90 22
e-mail: karin.ekberg@ks.se

Dr. Thomas Forst
Lilly Deutschland GmbH
SaalburgastraBe 153
D-61350 Bad Homburg, Germany
e-mail: forst_thomas@lilly.com

Dr. George Grunberger
Wayne State University
Director
Center for Molec Med & Genetics
540 E. Canfield Ave.
3216 Scott Hall
Detroit, MI 48201
Tel.: (313) 993-8029
Fax: (313) 577-5218
e-mail: ggrunberger@intmed.wayne.edu

Dr. Bo-Lennart Johansson
Karolinska Institutet
Karolinska Hospital
S-171 76 Stockholm, Sweden
Tel.: + 46 8 5177 5035
Fax: + 46 8 32 90 22
e-mail: bo-lennart.johansson@ks.se

Dr. Hans Jörnvall
Karolinska Institutet
Dept. of Med. Biochemistry and Biophysics
S-171 77 Stockholm, Sweden
Tel.: + 46 8 728 7702
Fax: + 46 8 33 74 62
e-mail: hans.jornvall@mbb.ki.se

Dr. Thomas Kunt
Krankenhaus SUW
Georg-Staab-Str. 3
76855 Annweiler, Germany

Dr. Zhen-guo Li
Center for Molecular Medicine and Genetics
540 E. Canfield Ave.
Scott Hall, Room 3216
Detroit, MI 48201
Tel.: (313) 577-1082
e-mail: zg_li@hotmail.com

Dr. Rudolf Rigler
Karolinska Institutet
Dept. of Med. Biochemistry and Biophysics
S-171 77 Stockholm, Sweden
Tel.: + 46 8 728 68 00
Fax: + 46 8 32 65 05
e-mail: rudolf.rigler@mbb.ki.se

Dr. Anders A. F. Sima (Chair)
Wayne State University
WSU Morris Hood Jr. Comprehensive
Diabetes Center
540 E. Canfield Ave.
Dept. of Pathology, Room 9374 Scott Hall
Detroit, MI 48201
Tel.: (313) 577-1150
Fax: (313) 577-0057
e-mail: asima@med.wayne.edu

Dr. Mats Sjöquist
Dept. of Physiology
Biomedicum, Box 572
S-751 23 Uppsala, Sweden
Tel.: + 46 18 471 4181
Fax: + 46 18 471 4938
e-mail: mats.sjoquist@physiology.uu.se

Dr. Donald F. Steiner
Howard Hughes Med Inst, N-216
5841 S Maryland Ave.
Chicago, IL 60637
Tel.: (773) 702-1334
Fax: (773) 702-4292
e-mail: dfsteiner@midway.uchicago.edu

Dr. Philippe Vague
CHU Timone
Service de Nutrition
Diabetologie-Endocrinologie
Boulevard Jean Moulin
F-13385 Marseille Cedex 5,
France

Dr. John Wahren
Karolinska Institutet
Dept. of Surgical Sciences
Section of Clin. Physiology
Karolinska Hospital
S 171 76 Stockholm, Sweden
Tel.: + 46 8 5177 4947
Fax: + 46 8 32 90 22
e-mail: john.wahren@ks.se

Dr. Zhihui Zhong
Karolinska Institutet
Dept. of Surgical Sciences
Section of Clin. Physiology
Karolinska Hospital
S 171 76 Stockholm, Sweden
Tel.: + 46 8 5177 4744
Fax: + 46 8 32 90 22
e-mail: zhihui.zhong@kirurgi.ki.se

### *References*

[1] Steiner, D., Cunningham, D., Spigelman, L., and Aten, B. (1967). Insulin biosynthesis: evidence for a precursor. *Science*, **157**, 697–700.

[2] Steiner, D. and Oyer, P. (1967). Biosynthesis of insulin and a probable precursor of insulin by a human islet cell adenoma. *Proc. Natl. Acad. Sci. USA*, **57**, 473–481.

[3] Rigler, R., Pramanik, A., Jönasson, P., Kratz, Jansson, O., Nygren, P.-Å., Ståhl, S., Ekberg, K., Johansson, B.-L., Uhlén, S., Uhlén, M., Jörnvall, H. and Wahren, J. (1999). Specific binding of proinsulin C-peptide to human membranes. *Proc. Natl. Acad. Sci. USA*, **96**, 1318–1323.

[4] Wahren, J., Ekberg, K., Johansson, J., Henriksson, M., Pramanik, A., Johansson, B.-L., Rigler, R. and Jörnvall, H. (2000). Role of C-peptide in human physiology. *Am. J. Physiol. Endocrinol. Metab.*, **278**, E759–E768.

[5] Guo, H.-E., Jardetzky, T. S., Garrett, T. P. J., Lane, W. S., Strominger, J. L. and Wiley, D. C. (1992). Different length peptides bind to HLA-Aw68 similarly at their ends but bulge out in the middle. *Nature*, **360**, 364–366.

[6] Brown, J. H., Jardetzky, T. S., Gorga, J. C., Stern, L. J., Urban, R. G., Strominger, J. L. and Wiley, D. C. (1993). Three-dimensional structure of the human class II histocompatibility antigen HLA-DR1. *Nature*, **364**, 33–39.

[7] Johansson, B.-L., Borg, K., Fernquist-Forbes, E., Kernell, A., Odergren, T. and Wahren, J. (2000). Beneficial effects of C-peptide on incipient nephropathy and neuropathy in patients with type 1 diabetes–a three-month study. *Diabetic Medicine*, **17**, 181–189.

[8] Grunberger, G., Qiang, X., Li, Z.-G. Mathews, S. T., Sbrissa, D., Shisheva, A. and Sima, A. A. F., Molecular basis for the insulinomimetic effects of C-peptide. *Diabetologia* (under revision).

[9] Johansson, B.-L., Sjöberg, S. and Wahren, J. (1992). The influence of human C-peptide on renal function and glucose utilization in type 1 (insulin-dependent) diabetic patients. *Diabetologia*, **35**, 121–128.

[10] Johansson, B.-L., Linde, B. and Wahren, J. (1992). Effects of C-peptide on blood-flow, capillary diffusion capacity and glucose utilization in the exercising forearm of type 1 (insulin-dependent) diabetic patients. *Diabetologia*, **35**, 1151–1158.

[11] Zierath, J., Handberg, A., Tally, H. and Wallberg-Henriksson, H. (1996). C-peptide stimulates glucose transport in isolated human skeletal muscle independent of insulin receptor and tyrosine kinase activation. *Diabetologia*, **39**, 306–313.

[12] Ohtomo, Y., Aperia, A., Sahlgren, B., Johansson, B.-L. and Wahren, J. (1996). C-peptide stimulates rat renal tubular $Na^+$, $K^+$-ATPase activity in synergism with neuropeptide Y. *Diabetologia*, **39**, 199–205.

[13] Ohtomo, Y., Bergman, T., Johansson, B.-L., Jörnvall, H. and Wahren, J. (1998). Differential effects of proinsulin C-peptide fragments on $Na^+$, $K^+$-ATPase activity of renal tubule segments. *Diabetologia*, **41**, 287–291.

[14] Forst, T., DeLa Tour, D. D., Kunt, T., Pfützner, A. K., Pohlmann, T., Schneider, S., Johansson, B.-L., Wahren, J., Lobig, M., Engelbach, M., Beyer, J. and Vague, P. (2000). Effects of proinsulin C-peptide on nitric oxide, microvascular blood-flow and erythrocyte $Na^+$, $K^+$-ATPase activity in diabetes mellitus type 1. *Clin. Sci.*, **98**, 283–290.

[15] Sima, A. A. F., Yorek, M., Pierson, C. R., Murakawa, Y., Breidenbach, A. and Zhang, W. (2001). Dose dependent prevention of early neuropathy in the BB/Wor-rat with human C-peptide. *American Diabetes Association*. Philadelphia.

[16] Ido, Y., Vindigni, A., Chang, K., Stramm, L., Chance, R., Heath, W., DiMarchi, R., Cera, E. D. and Williamson, J. (1997). Prevention of vascular and neural dysfunction in diabetic rats by C-peptide. *Science*, **277**, 563–566.

[17] Wahren, J., Owad, S., Johansson, B.-L., Berggren, P.-O. Bertorello, A. (1996). C-peptide stimulates $\beta$-cell $Na^+$, $K^+$-ATPase activity a negative feedback mechanism on insulin secretion? *Diabetologia*, **39** (suppl. 1), A71.

[18] Kunt, T., Schneider, S., Pfützner, A. K., Engelbach, M., Shant, B., Beyer, J. and Forst, T. (1999). The effect of human proinsulin C-peptide on erythrocyte deformability in patients with type 1 diabetes mellitus. *Diabetologia*, **42**, 465–471.

[19] Sima, A. A. F., Zhang, W., Sugimoto, K., Henry, D., Li, Z.-G., Wahren, J. and Grunberger, G., C-peptide prevents and improves chronic type 1 diabetic neuropathy in the BB/Wor-rat. *Diabetologia* (in press).

[20] Sugimoto, K., Murakawa, Y., Zhang, W., Xu, G. and Sima A. A. F. (2000). Insulin receptor in rat peripheral nerve: its- localization and alternatively spliced isoforms. *Diab. Metab. Res. and Rev.*, **16**, 354–363.

[21] Li, Z.-G., Zhang, W., Grunberger, G. and Sima, A. A. F. (2000). C-peptide corrects IGF's and prevents apoptosis in type 1 BB/W-rat hippocampus. *Diabetes*, **49** (suppl. 1), A229.

[22] Johansson, B.-L., Borg, K., Fernquist-Forbes, E., Odergren, T., Remahl, S. and Wahren, J. (1996). C-peptide improves autonomic nerve function in patients with type 1 diabetes. *Diabetologia*, **39**, 687–695.

[23] Kunt, T., Forst, T., Closs, E., Wallerath, U., Förstermann, R., Lehmann, R., Pfützner, A., Harzer, O., Engelbach, M. and Beyer, J. (1998). Activation of endothelial nitric oxide synthase (eNOS) by C-peptide. *Diabetologia*, **41**, A176.

[24] Johansson, B. L., Pernow, J. and Wahren, J. (1999). Muscle vasodilatation by C-peptide is NO-mediated. *Diabetologia*, **42**, A324.

[25] Jensen, M. E. and Messina, E. J. (1999). C-peptide induces a concentration-dependent dilatation of skeletal muscle arterioles only in presence of insulin. *Am. J. Physiol.*, **276**, H1223–H1228.

[26] Sjöqvist, M., Huang, W. and Johansson, B.-L. (1998). Effects of C-peptide on renal function at the early stage of experimental diabetes. *Kidney International*, **54**, 758–764.

[27] Samnegård, B., Jacobson, S., Jaremko, G., Johansson, B.-L. and Sjökvist, M. (2001). Effects of C-peptide on renal function, albuminuria and glomerular volume in diabetic rats. *Kidney International*, submitted.

[28] Johansson, B. L., Kernell, A., Sjöberg, S. and Wahren, J. (1993). Influence of combined C-peptide and insulin administration on renal function and metabolic control in diabetes type 1. *J. Clin. Endocrin. Metab.*, **77**, 976–981.

[29] Lindström, K., Johansson, C., Johnsson, E. and Haraldsson, B. (1996). Acute effects of C-peptide on the microvasculature of isolated perfused skeletal muscle and kidneys in rat. *Acta Physiol. Scand.*, **156**, 19–25.

